# The effect of long-term confinement and the efficacy of exercise countermeasures on muscle strength during a simulated mission to Mars: data from the Mars500 study

**DOI:** 10.1186/s40798-017-0107-y

**Published:** 2017-11-13

**Authors:** Christopher J. Gaffney, Elena Fomina, Dennis Babich, Vladimir Kitov, Konstantin Uskov, David A. Green

**Affiliations:** 1 Institute of Biomedical Problems (IBMP), Moscow, Russia; 2 Centre of Human & Aerospace Physiological Sciences (CHAPS), King’s College London, Faculty of Life Sciences & Medicine, Guy’s Campus, London, SE1 1UL UK; 3 KBRwyle, European Astronaut Centre, Linder Höhe, D-51147 Cologne, Germany

**Keywords:** Mars500, Confinement, Muscle strength, Spaceflight, Ground-based analogue, Spaceflight analogue, Intervention

## Abstract

**Background:**

Isolation and long duration spaceflight are associated with musculoskeletal deconditioning. Mars500 was a unique, high-fidelity analogue of the psychological challenges of a 520-day manned mission to Mars. We aimed to explore the effect of musculoskeletal deconditioning on three outcome measures: (1) if lower limb muscle strength was reduced during the 520-day isolation; (2) if type I or II muscle fibres were differentially affected; and (3) whether any 70-day exercise interventions prevented any isolation-induced loss of strength.

**Methods:**

Six healthy male subjects (mean ± SEM) (34 ± 3 years; 1.76 ± 0.02 metres; 83.7 ± 4.8 kg) provided written, informed consent to participate. The subjects’ maximal voluntary contraction (MVC) was assessed isometrically in the calf (predominantly type I fibres), and maximal voluntary isokinetic force (MVIF) was assessed in the quadriceps/hamstrings (predominantly type II fibres) at 0.2 and 0.4 ms^−1^ using the Multifunctional Dynamometer for Space (MDS) at 35-day intervals throughout Mars500. Exercise interventions were completed 3–7 days/week throughout the 520-day isolation in a counterbalanced design excluding 142–177 days (rest period) and 251–284 days (simulated Mars landing). Exercise interventions included motorized treadmill running, non-motorized treadmill running, cycle ergometry, elastomer-based resistance exercise, whole-body vibration (WBV), and resistance exercise using MDS.

**Results:**

Calf MVC did not reduce across the 520-day isolation and MDS increased strength by 18% compared to before that of 70-day exercise intervention. In contrast, there was a significant bilateral loss of MVIF across the 520 days at both 0.2 ms^−1^ (*R* ^2^ = 0.53; *P* = 0.001) and 0.4 ms^−1^ (0.4 ms^−1^; *R* ^2^ = 0.42; *P* = 0.007). WBV (+ 3.7 and 8.8%) and MDS (+ 4.9 and 5.2%) afforded the best protection against isolation-induced loss of MVIF, although MDS was the only intervention to prevent bilateral loss of calf MVC and leg MVIF at 0.2 and 0.4 ms^−1^.

**Conclusions:**

Mars500 induced significant loss of quadriceps/hamstrings MVIF but not calf MVC. Collectively, these data suggest that muscles with predominantly type I fibres were affected less by isolation compared to type II dominant muscles. MDS and WBV afforded the best protection against isolation-induced loss of strength and thus may have virtue in exploration class missions.

**Electronic supplementary material:**

The online version of this article (10.1186/s40798-017-0107-y) contains supplementary material, which is available to authorized users.

## Key points

- Mars500 was the longest isolation study ever conducted in humans and shows that despite exercising on a regular basis, crew lost significant leg strength.
- Habitual physical activity is therefore critical to strength maintenance.
- This has great implications for spaceflight and also for public health initiatives.

## Background

Exploration of Mars will require astronauts to remain healthy in space for longer than previously achieved [1] as missions to Mars are proposed to be in excess of a year [1, 2]. However, astronauts must be able to perform adequately, if not optimally, on Mars, where the gravity is ≈ 38% (3.711 ms^−2^) of that on Earth [3]. Losses in strength of up to 50% are predicted for a mission to Mars [4], which is equivalent to that associated with ageing from the second to the seventh decade of life [5, 6]. Strength loss therefore represents a serious concern for astronaut health, and intervention data are needed to determine the best protection for astronauts against microgravity.

Similarly to spaceflight, ground-based analogue studies have shown that prolonged confinement (e.g. in submariners) can result in loss of muscle strength in only 2 months [7]. Furthermore, confinement [8] and sustained operational stress is sufficient to induce muscle atrophy in young, healthy, active, and relatively well-nourished individuals [9]. It is not known, however, if a simulated Mars mission can induce muscle atrophy and associated losses in strength despite the presence of gravitational loading and whether exercise interventions can protect against any loss. It is also not known if starting strength [10] or pre-isolation training volume [11] have any affect on the strength lost during long-term isolation or spaceflight. Certainly, lack of exercise compliance has been cited as an explanation for loss of strength encountered in early ISS missions compared to previous Russian space station missions [12]. Mars500, conducted at the Institute of Biomedical Problems (IBMP) in Moscow, attempted to simulate some of the psychological and physiological challenges of a 520-day manned mission to Mars by isolating a crew in a hermetically sealed, confined, spacecraft-like environment. Whilst not simulating microgravity, Mars500 sought to emulate the isolation, boredom, controlled diet, relative inactivity, and stress managing finite resources autonomously with only limited and delayed communication with ‘the ground’ (mars500.ibmp.ru).

Spaceflight is associated with a preferential loss of type I muscle fibres [11, 13, 14] and muscle atrophy [15], which is associated with reduced strength (maximum voluntary contractions (MVC)) [11]. It not known if this is similar with long-term isolation. To investigate whether isolation causes preferential atrophy of type I or II fibre dominant muscles, isokinetic dynamometry can be employed to differentiate strength in muscles containing different proportions of slow and fast contracting fibres [16, 17, 18]. In the calf, there are up to double the quantity of type I muscle fibres (soleus ~ 70–80%; gastrocnemius ~ 50–57%) known to be preferentially lost in spaceflight [14] than in the thigh (vastus lateralis ~ 32–42%; biceps femoris ~ 47%) [19–22].

Despite decades of human spaceflight, optimal lower limb exercise interventions remain to be determined. Cycle ergometry with a Vibration Isolation and Stabilisation System (CEVIS) using loads of 25–350 W and treadmill running with a vibration isolation system (TVIS) (a treadmill with a harness to secure the user and mimic gravitational loading) are routinely employed but cannot prevent loss of calf and thigh muscle volume during 6-month ISS missions [23]. Resistance exercise may curtail muscle atrophy [24], thereby reducing task-specific force/power loss via changes upstream of the neuromuscular junction [16]. Resistance exercise may also alter the hormonal milieu with exercise to prevent muscle catabolism [17] and promote the maintenance of muscle protein synthesis [25]. Indeed, the advanced resistive exercise device (ARED) on the ISS appears promising [26] but it is unsuitable for exploration class missions because of size/mass. The Multifunctional Dynamometer for Application in Space (MDS) is smaller in size and mass than ARED and can be used to assess lower limb muscle strength. Furthermore, it can be used for exercise interventions including squat, bench press, dead lift, lateral pull, back extension, and rowing, in addition to calf raises and leg presses. WBV enhances motor unit recruitment promoting an efficient, specific warm-up effect that allows the muscle to produce more force and power and aids flexibility [27, 28]. Resistance exercise coupled with vibration (WBV) has prevented muscle atrophy during 56-day bed rest [29] and may provide a simple intervention [28] suitable for exploration class missions. Furthermore, elastic expanders train many muscle groups and have low mass making them viable for long-term missions [30]. It is not known, however, if these interventions can prevent isolation-induced lower-limb loss of strength. Thus, the study sought to test the hypotheses that (1) the 520-day isolation during Mars500 was sufficient to induce loss of strength in the calf and quadriceps/hamstrings muscles; (2) muscles comprising largely of type I muscle fibres were preferentially affected; (3) loss of strength was associated with strength before Mars500; and (4) the performance of 70-day exercise interventions including the MDS and WBV were able to prevent any loss of strength.

## Methods

### Subjects

Six healthy male participants (34 ± 3 years; 1.76 ± 0.02 metres; 83.7 ± 4.8 kg) gave written informed consent to participate in the study that conformed to the 6th revision of the *Declaration of Helsinki* and had received ethics approval from the local university and the Institutional Review Board of the Institute for Biomedical Problems (IBMP).

Three Russian, two European, and one Chinese crew member were isolated for a total of 520 days within the habitable module of the Mars500 facility located at the IBMP. The experiment was completed under normal (Earth) gravitational conditions. All subjects completed a medical screening, which included an ECG at rest and during exercise, chest and spine X-rays, ultrasound assessment of internal organs, and blood tests to test for any inability to complete the 520-day isolation experiment an exercise training during the study. Subjects were psychologically screened for social compatibility and motivation via the Minnesota Multiphasic Personality Inventory [31].

### Experimental protocol

During the 520-day isolation, the subjects were instructed to follow an exercise intervention timetable to prevent loss of strength during isolation. The subjects were randomly grouped into pairs (1 and 5, 2 and 6, 3 and 4) and all pairs completed every exercise intervention but at different dates during isolation, in a counterbalanced design. The timetable can be found in [32]. Subjects 1 and 5 correspond to ‘O’ and ‘N’, 3 and 4 to ‘M’ and ‘L’, and 2 and 6 to ‘I’ and ‘K’ in [32]. The subjects completed different exercise interventions every 70 days, and these interventions comprised of active running, passive running, cycle ergometry, expanders, and WBV or MDS. Exercise interventions were not completed during 142 to 177 days (rest period) and 251 to 284 days (simulated landing activities on Mars [33]). Heart rate (Polar RS400 monitor, Kempele, Finland) was recorded throughout all training sessions to ensure participant safety.

### Endurance exercises—treadmill running and cycle ergometry

Endurance exercise interventions to prevent loss of strength comprised of active (motorized) treadmill running, passive mode (non-motorized) treadmill running, and cycling, all completed 5–6 days/week for each 70-day training block. Active and passive mode treadmill running were completed on a Cybex International 750T treadmill (Medway, MA, USA) and a non-commercial treadmill (BD-1) used in the Russian module of the International Space Station, following the training protocol in Additional file 1: Figure S1 A–C.

Training on the cycle ergometer was completed on a Kettler Velergometer (Ense, Germany), following the training protocol in Additional file 1: Figure S1 D–F. Subjects were instructed to maintain a cadence of 60–70 rpm whilst cycling. All endurance mode exercises were completed as 3 days training followed by 1 day of rest (training therefore 5–6 days/week throughout the 70-day block).

### Resistance exercise—expanders, WBV, and MDS

Resistance exercise interventions to prevent loss of strength comprised of expanders, WBV, and MDS. Before completing expander training (elastomer-based resistance exercise), the subjects completed a non-prescriptive 10-min warm-up comprising of low-velocity closed-chain movements (e.g. squats) and passive stretching (e.g. quadriceps, hamstrings, and calf stretches). Subjects completed three different exercise protocols using expanders weekly, throughout the 70-day training block (total training of 3–4 days/week). Protocol 1 comprised of 2 × 15–20 reps of elbow flexion, calf raises, abdominal crunches, and standing trunk extensions; protocol 2 comprised of 2 × 15–20 reps of press ups, squats, rowing, and pull downs; protocol 3 (recovery) comprised of three to five hamstring stretches in two positions. Training sessions were completed every other day during the 70-day training block (3–4 days/week).

Exercise using WBV was completed on a Galileo oscillating platform (Novotec Medical-Stratec Medizintechnik, Germany) at amplitudes of 2–6 mm depending upon foot position, and at a fixed range of frequencies from 16 to 25 Hz (Table 1). Subjects were familiarised with exercise and foot placement during WBV; however, data on foot position was not collected and foot position was not enforced during exercise. Subjects completed protocol A 5 days/week (Tuesday, Wednesday, Friday, Saturday, and Sunday), at 9.30 a.m. and 5.30 p.m., at least an hour after breakfast and dinner. Subjects completed protocol B 2 days/week on the remaining days (Monday and Thursday), in a single 11-min session (Table 1).

**Table 1:** Exercise protocols A and B performed during WBV

Time (min)	Frequency (Hz)	Exercise
Protocol A		
1	16–18	Warm-up
2–4	16–18	Rapid shallow sit ups
5	16–18	Squats (4 s down; 4 s up)
6	16–18	Two-footed vertical jumps on the balls of feet
7–8		*Break*
9–10	16–18	Pelvis movements (alternating hip flexion and extension)
11	16–18	Maximal hip flexion
12	16–18	Maximal hyperextension
13	16–18	Hands behind head and twist the body maximally clockwise, then anti-clockwise
14	16–18	Hip adduction reaching towards the ankle with the hand whilst the other hand reaches into the air. Movement is only in the frontal plane
Protocol B*		
1	16–18	Warm-up
2	24–25	Rapid shallow sit ups
3	24–25	Basic stance
4–6	24–25	Deep squat
7–9		*Break*
10–11	24–25	Basic stance. Deep prised (posture skier) within a maximum time
12–13	24–25	Heels keep
14–16		*Break*
17–18	24–25	Basic stance (24–25 Hz) ‘Lifting the socks’ action
Example warm-up		
1	16–18	Crew member stands in a vertical position (with slightly bent knees, and the feet in the basic stance)

*20 kg tabard is worn throughout all Protocol B exercises

Exercise on MDS (Vienna University of Technology, Austria) comprised of three to five sets of 10–20 reps of squats, calf raises, sit ups, seated row, lateral pull back, bench press, and neck muscle exercises completed every other day (3–4 days/week) (Additional file 2: Table S1).

### Strength assessments

Strength assessments were completed at least 24 h after the previous training session to allow sufficient rest. MVC of the calf working isometrically was determined at 90° plantar flexion whilst subjects were seated on MDS. Maximal voluntary isokinetic force (MVIF) was determined for leg press separately in the left and right legs at 0.2 and 0.4 ms^−1^, respectively, and the mean average was calculated to determine bilateral leg strength. Isometric assessment was completed for the calf since it generates greater force [34] and isokinetic assessment was utilized for quadriceps/hamstring strength since it is the more sensitive at detecting changes in strength than isometric assessment [35]. The leg press was completed in the seated position, and the subjects were required to extend the hip and knee joints to push the resistance away from the body at either 0.2 or 0.4 ms^−1^. Force was measured using MDS and visual data on force produced was fed back immediately to the subject via a digital transducer to assist the subjects with the maintenance of effort (Vienna University of Technology, Vienna, Austria). Subjects completed three maximal isometric (calf) or isokinetic (quadriceps/hamstrings) contractions with approximately 60 s rest between trials. The greatest was taken as the MVC or MVIF. Strength assessments (MVC and MVIF) were completed before the start of Mars500, the day of entering isolation, and every 35 days throughout the 520-day isolation, capturing the period immediately before and at the end of each 70-day exercise intervention.

Previous work has shown that the proportion of type II (fast twitch) muscle fibres (determined by mATPase staining) shows a moderate-high correlation with MVIF at medium contractile velocities (*r* ^2^ = 0.75) and a high correlation with MVIF at high contraction velocities (*r* ^2^ = 0.89; [36]). The assessment of MVIF using isokinetic dynamometry on MDS at both 0.2 and 0.4 ms^−1^ can therefore be used to non-invasively determine changes in type II muscle fibres over the Mars500 study.

### Data analysis

A power calculation was completed using data from a previous long-term spaceflight experiment [37]. Data demonstrated that long-term spaceflight (6 months) induced a loss of strength of 31% in the lower limb muscles, the same muscle group we are studying. Given that loss of strength and muscle mass in spaceflight is roughly three times greater than in ground-based analogues, we used these data to estimate the potential loss of strength during 18 months confinement, for which there are no data to base a power calculation. Using these data [37] to calculate an effect size (dz) of 1.63 and using an alpha of 0.05 and a power of 0.95, we get a minimum sample required of *n* = 6 for detecting a difference in strength, which was the primary outcome of our study. Indeed, in our regression analysis, we have shown significant loss of strength over the 520-day isolation (*P* = 0.001 for 0.2 ms^−1^ and *P* = 0.007 for 0.4 ms^−1^), and therefore, our study was appropriately powered.

Training volume on MDS was calculated to investigate the effect of total training volume on the preservation of strength during 70-day training with MDS. Force and position data was recorded continuously at 50 Hz during calf and leg press training sessions on MDS. Data were processed using customized scripts in MATLAB® (R2010b, The MathWorks, Inc.®, Natick, MA, USA) that calculated total training volume from number of repetitions and force per repetition.

Data were checked to ensure no statistical assumptions were violated (autocorrelation assessed using scatter plot of residuals), and linear regression was used to determine the change in calf MVC and bilateral leg MVIF from 0 day and every 35 days throughout the 520-day isolation. Paired *t* tests were also completed between MVC/MVIF at 0 and 520 days, and 35 and 520 days to determine whether isolation significantly reduced strength. To determine whether starting strength determined change in strength during isolation, linear regression was completed between starting MVC/MVIF and change in MVC/MVIF throughout the 520-day isolation. All data were analysed using GraphPad Prism (USA) with statistical significance defined as *P* < 0.05 although due to the low *n* number, changes in MVC/MVIF > ± 10% were deemed functionally significant.

## Results

### Effect of Mars500 upon calf raise MVC and leg press MVIF

**Fig. 1:**
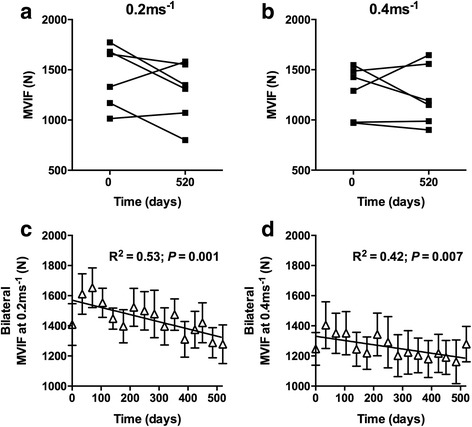
Mean (±SEM) bilateral leg press maximal voluntary isokinetic force (MVIF) at 0.2 and 0.4 ms^−1^ reduced with time during Mars500. Bilateral MVIF was measured every 35 ± 3.6 days in each subject during the 520-day isolation period at 0.2 and 0.4 ms^−1^ contraction rates. Individual (**a**, **b**) and mean (±SEM) (**c**, **d**) MVIF forces at 0.2 and 0.4 ms^−1^ are shown respectively

Calf raise MVC did not reduce across isolation but rather increased non-significantly (0 vs. 520 days; *P* > 0.05) by 7.6% from 1641 ± 113 N to 1766 ± 82 N. In contrast, there was a bilateral and relatively linear significant loss of strength at both low (0.2 ms^−1^; *R* ^2^ = 0.53; *P* = 0.001) and high velocity (0.4 ms^−1^; *R* ^2^ = 0.42; *P* = 0.007) isokinetic leg presses across 520 days (Fig. 1a). The change in strength at 0.2 and 0.4 ms^−1^ did not reach statistical significance using a pre- vs. post-*t* test (0 vs. 520 days; *P* > 0.05). However, 0 day is not representative of normal strength, for example, due to detraining pre-spaceflight that occurs during the quarantine period, hence the significant increase in strength at 35 days (Fig. 1c, d). A *t* test between 35 and 520 days did confirm a significant reduction in strength across time at 0.2 ms^−1^ (*P* = 0.001) and 0.4 ms^−1^ (*P* = 0.02).

### Relationship between pre-isolation MVC and changes induced by Mars500

**Fig. 2:**
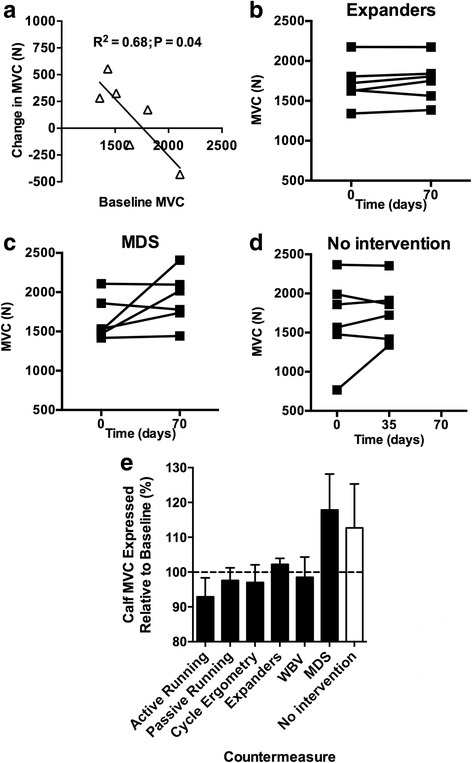
**a** Change in calf MVC across Mars500 was significantly associated with starting calf MVC. Calf MVC was measured every 35 ± 3.6 days in each subject during the 520-day isolation period. **b**, **c** Individual changes in MVC where the intervention on average protected against loss of strength and **d** where no exercise intervention was completed. **e** Mean (±SEM) calf MVC was increased relative to baseline (day 0) following candidate 70-day exercise interventions

Pre-isolation calf MVC was significantly negatively correlated (*R* ^2^ = 0.684; *P* = 0.042) with change in MVC across the 520 days (Fig. 2), although this includes four subjects whom increased calf MVC. In contrast, reductions in MVC at 0.2 ms^−1^ (*R* ^2^ = 0.237; *P* = 0.327) and 0.4 ms^−1^ (*R* ^2^ = 0.061; *P* = 0.637) did not correlate with pre-isolation values.

### Intervention efficacy in ameliorating Mars500-induced loss of calf MVC

There was no significant reduction in calf MVC across the 520 days (*R* ^2^ = 0.04; *P* = 0.46), although there were moderate increases observed (with respect to 0 day) following 70 days use of expanders (2.2 ± 1.7%) and MDS (17.8 ± 10.6) (Fig. 2). Moreover, a 12.7% increase was observed during 35 days of no exercise intervention (Fig. 2). Seventy days of active running (− 7.1 ± 5.5%), passive running (− 2.4 ± 3.7), cycle ergometry (− 3.0 ± 5.2%), and WBV (− 1.5 ± 6.0%) failed to protect against loss of MVC during the 70-day exercise intervention.

### Intervention efficacy in ameliorating Mars500-induced loss of leg press MVIF

**Fig. 3:**
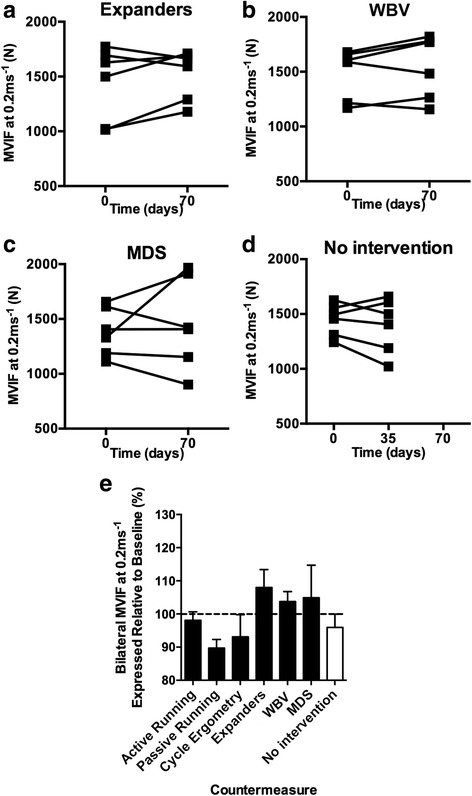
**a**–**c** Individual data from exercise interventions that on average protected against loss of strength at 0.2 ms^−1^ and **d** where no exercise intervention was completed. **e** Mean (±SEM) bilateral leg MVIF at 0.2 ms^−1^ change following candidate 70-day exercise interventions

**Fig. 4:**
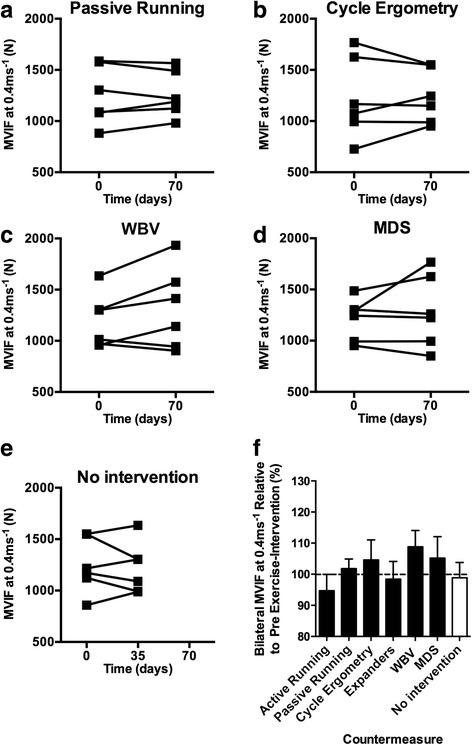
**a**–**d** Individual data from exercise interventions that on average protected against loss of strength at 0.4 ms^−1^ and **e** where no exercise intervention was completed. **f** Mean (±SEM) bilateral leg MVIF at 0.4 ms^−1^ change following candidate 70-day exercise interventions

The 35-day time point of no exercise intervention was associated with a 4.0 ± 4.1% loss of MVIF at 0.2 ms^−1^ (Fig. 3). At 0.2 ms^−1^, active running, passive running, and cycle ergometry all failed to protect against loss of MVIF (Fig. 3). Expanders (8.0 ± 5.5%), WBV (3.7 ± 3.1%), and MDS (4.9 ± 10.0%) protected against loss of strength and increased strength (albeit < 10%) vs. before the 70-day exercise intervention. The 35-day time point of no exercise intervention was associated with a 1.1 ± 5.0% loss of MVIF (Fig. 4). At 0.4 ms^−1^, MVIF was not protected by active running or expanders. WBV (8.8 ± 5.4%) and MDS (5.2 ± 7.0%) were the most effective interventions at 0.4 ms^−1^ (Fig. 4).

## Discussion

The main findings of this study were that the 520-day Mars500 isolation mission did not induce loss of calf MVC. In contrast, significant reductions in bilateral MVIF at both 0.2 and 0.4 ms^−1^ were observed that were not related to pre-isolation MVIF. At 0.2 ms^−1^, expanders were the most effective intervention, inducing increased MVIF along with WBV and MDS. WBV and MDS were also the most effective interventions to prevent loss of MVIF at 0.4 ms^−1^.

Significant reductions in MVIF were observed at 0.2 and 0.4 ms^−1^ across Mars500 that were not evident for calf MVC. This contrasts with calf MVC reductions being frequently associated with long-term spaceflight [11, 13]. Indeed, during 35 days of no intervention, calf MVC actually increased (+ 12.7%). Mars500, however, involved postural loading unlike microgravity, which perhaps provided sufficient stimulus to prevent loss of strength, although interpretation of data from so few subjects is challenging.

Preferential type I muscle fibre atrophy is associated with microgravity and disuse [38]. Data from this study show that strength was lost in the quadriceps/hamstrings but not the calf muscles. The calf contains a high proportion of type I fibres and the vastus lateralis (quadriceps) and biceps femoris (hamstrings) contain a higher proportion of type II fibres [19–22]. Furthermore, determination of MVIF using MDS is a high-fidelity assessment of type II muscle fibres [36]. Collectively, the preservation of MVC in the calf and the loss of MVIF across the 520 days in the quadriceps/hamstrings suggest loss of strength in type II but not type I muscle fibres.

If the magnitude of losses observed during Mars500 is representative of strength losses on the Martian surface, they could have a significant negative impact upon mission success. Whilst not assessed in this study, on Earth, leg strength is positively correlated with walking speed [39], mobility [40], and reduced lower muscle strength; in particular, the calf is associated with increased falls [41] and subsequent hip fracture risk [42]. Thus, mechanistic understanding to inform effective interventions are required for Earth and exploration missions such as to Mars [1, 12].

Such strength losses, observed despite periodic intervention performance comparable to those employed on-board the ISS, suggests that the effect of isolation is substantial and warrants further investigation to determine the role of isolation itself [8], nutrition [7], and operational stress [9]. Nevertheless, the loss of MVIF in otherwise healthy individuals suggests that Mars500 was a valid isolation model comparable to other spaceflight analogues [7] and may have implications for long-term spaceflight recommendations that previously focused on microgravity as the cause of muscle atrophy [43]. Prolonged confinement may reduce exercise motivation [8] over time, which could be exaggerated as a crew reduces contact with mission control and the apparent ‘need’ to exercise in microgravity is unclear. Indeed, substantial negative changes in body composition, cardiovascular de-conditioning, bone, and strength losses have been observed in submarine and Antarctic overwinter crews [44]. The Mars500 participants were provided with an artificial light-dark cycle comparable to the ISS which itself can reduce vitamin D levels [45], which is associated with lower isometric leg strength [46].

Interestingly, the loss of leg press MVIF was not related to initial MVIF. One subject lost 369 N (− 22%) in strength vs. 0 day, and this loss of strength was equivalent to ageing from the second to seventh decade of life [6]. However, given the low *n* number and the potential differential sensitivity to isolation vs. unloading in a given individual, further investigation is warranted to determine whether pre-mission physical training is advantageous for long-term space missions [10]. Indeed, further studies should also examine the relationship between the familiarity with exercise pre-isolation/spaceflight and the individual response to particular exercises during isolation/spaceflight.

The failure to observe a significant reduction in calf MVC over the 520-day mission may relate to the subjects’ ability to ambulate and weight-bear but could also reflect, at least in part, effective engagement in exercise interventions. As a result, whilst potentially ethically challenging, a control group that do not participate in any exercise throughout the 520 days is warranted to enable further comparison with bed rest studies [47], although not spaceflight studies. It must also be noted that calf strength was determined isometrically and quadriceps/hamstring strength isokinetically. Not determining strength in an identical mode is a study limitation and perhaps contributes to the differences observed in different muscle groups.

During Mars500, a range of current and candidate exercise interventions for an exploration class mission were employed for 70-day periods at training volumes considered in excess of those required to have a positive effect. This appears the case as moderate increments in calf MVC were noted following use of expanders and MDS. MDS [48] produced the greatest protection against loss of lower-limb strength but whether its employment is warranted in addition to, or instead of running [49] or cycling [23], currently key components of the astronaut health maintenance system needs to be tested.

In contrast, 70 days active running, passive running, and cycle ergometry failed to protect against Mars500-induced reduction in 0.2 ms^−1^ MVIF. However, the use of expanders, WBV, and MDS were not only protective but induced small increases in MVIF. The better protection afforded by WBV at 0.4 ms^−1^ complements the shift in the velocity-force curve [50] to the right (i.e. faster) [27] previously observed. In fact, all exercise interventions with the exception of active running (− 5.3 ± 5.4%) and expanders (− 1.6 ± 5.8%) were able to protect against loss of strength during the 70-day exercise intervention. Some differences in exercise efficacy were perhaps attributable to total training volume. The more effective exercise interventions such as MDS (3–4 days/week) and WBV (7 days/week) had higher training volumes, whereas the less effective exercises had lower training volumes in some instances (e.g. expanders 3 days/week) but not others (e.g. active running 5–6 days/week). Future isolation and spaceflight studies should match exercise interventions for total energy expenditure to increase comparability and remove this limitation.

Taken together, data from within our unique, but small cohort, provides evidence to support the proposition that the MDS [48] and exercise during WBV [29] are the most promising interventions for maintenance of calf and leg strength [30]. However, neither should be viewed as replacements for resistance exercise (via advanced resistive exercise device (ARED)) [24], currently considered the optimal ISS countermeasure [26]. ARED is impractical for exploration class missions due to its mass and volume, but similar equipment capable of multi-muscle comparison and the determination of optimal training volume should be tested in future studies.

## Conclusions

Despite not unloading participants, the Mars500 analogue induced significant loss of MVIF in the legs, which were unrelated to pre-isolation MVIF with little evidence for preferential type I fibre loss. Investigation of the factors that induce such losses is warranted. No significant losses in calf MVC were observed, but this presumably relates to continued weight-bearing activity. MDS and WBV were the most effective candidate interventions in ameliorating strength loses and thus may have virtue in exploration class missions as they are less complex and resource-intensive than ARED, although optimal training volume and adherence remain to be determined.

## Additional files

Additional file 1: Figure S1. Treadmill: (A–C) Treadmill protocol for day 1 (A), day 2 (B), and day 3 (C). Areas where the line is 0 km/h denote pre-defined breaks in the profile. Cycling: (D–F) Profiles for days 1 (D), 2 (E), and 3 (F) on the cycle ergometer. Different profiles provided variability to maximize potential physiological adaptation and prevent boredom, thereby aiding compliance. (PNG 138 kb)

Additional file 2: Table S1. Multifunctional Dynamometer for Application in Space (MDS) exercise protocol (DOCX 11 kb)
