# Supplementary figures and images for: The effect of long-term confinement and the efficacy of exercise countermeasures on muscle strength during a simulated mission to Mars: data from the Mars500 study
Source: Sports Med Open. 2017 Nov 13;3:40. doi: 10.1186/s40798-017-0107-y (PMC5684057; doi:10.1186/s40798-017-0107-y)

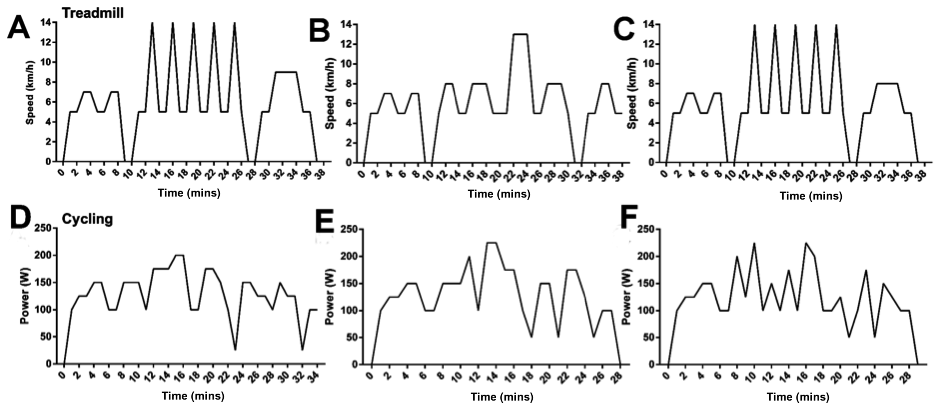

Supplement: Supplementary file 1 — Treadmill: (A–C) Treadmill protocol for day 1 (A), day 2 (B), and day 3 (C). Areas where the line is 0 km/h denote pre-defined breaks in the profile. Cycling: (D–F) Profiles for days 1 (D), 2 (E), and 3 (F) on the cycle ergometer. Different profiles provided variability to maximize potential physiological adaptation and prevent boredom, thereby aiding compliance. (PNG 138 kb) [file 40798_2017_107_MOESM1_ESM.png]
