# Supplementary material for: The effect of long-term confinement and the efficacy of exercise countermeasures on muscle strength during a simulated mission to Mars: data from the Mars500 study
Source: Sports Med Open. 2017 Nov 13;3:40. doi: 10.1186/s40798-017-0107-y (PMC5684057; doi:10.1186/s40798-017-0107-y)
Supplement: Supplementary file 2 — Multifunctional Dynamometer for Application in Space (MDS) exercise protocol (DOCX 11 kb) [file 40798_2017_107_MOESM2_ESM.docx]

**Additional file 2: Table S1.** Multifunctional Dynamometer for Application in Space (MDS) Exercise Protocol

| **Exercise Number** | **Exercise** | **Sets** | **Repetitions** |
| --- | --- | --- | --- |
| ***Day 1*** | | | |
| 1 | Squats | 5 | 12 - 15 |
| 2 | Standing Calf Raises | 3 | 15 - 20 |
| 3 | Full inclination sit-ups from supine position | 3 | 15 - 20 |
| 4 | Exercises for neck muscles with expanders | 5 | 15 - 20 |
| ***Day 2*** | | | |
| 1 | Seated row | 4 | 10 - 12 |
| 2 | Standing lateral pull-back | 4 | 10 - 15 |
| 3 | Standing Calf Raises | 3 | 20 - 25 |
| 4 | Exercises for neck muscles with expanders | 5 | 15 - 20 |
| ***Day 3*** | | | |
| 1 | Bench Press | 4 | 10 - 12 |
| 2 | Standing Calf Raises | 4 | 15 - 20 |
| 3 | Full inclination sit-ups from supine position | 5 | 15 - 20 |
| 4 | Exercises for neck muscles with expanders | 5 | 15 - 20 |
